# Thyroid Hormone in Hepatocellular Carcinoma: Cancer Risk, Growth Regulation, and Anticancer Drug Resistance

**DOI:** 10.3389/fmed.2020.00174

**Published:** 2020-05-22

**Authors:** Yang-Hsiang Lin, Kwang-Huei Lin, Chau-Ting Yeh

**Affiliations:** ^1^Liver Research Center, Chang Gung Memorial Hospital, Taoyuan, Taiwan; ^2^Department of Biochemistry, College of Medicine, Chang Gung University, Taoyuan, Taiwan

**Keywords:** thyroid hormone, thyroid hormone receptor, hypothyroidism, therapeutic target, hepatocellular carcinoma

## Abstract

Thyroid hormone (TH) and its receptor (TR) are involved in differentiation, metabolic process, and growth regulation in hepatocellular carcinoma (HCC). The TH/TR complexes are ligand-dependent transcriptional factors, functioning through binding to thyroid hormone response elements (TREs) upstream of the target genes. To date, deciphering the biological effects of TH in cancer progression remains challenging. Several lines of evidence suggest a growth inhibitory effect of TH in liver cancer. Mutation and aberrant expression of TRs are highly correlated with several types of cancers including HCC. Several reports show that TH inhibits cell growth in liver cancer through regulation of cell-cycle-related genes and non-coding RNAs. A case–control study indicates that hypothyroidism is associated with an increased risk of HCC. Moreover, TH/TR suppresses hepatocarcinogenesis via selective autophagy. Conversely, other groups have indicated that TH promotes cancer cell proliferation. *In vitro* and *in vivo* experiments show that TH/TR enhances cancer cell migration and invasion, anticancer drug resistance, angiogenesis, and cancer stem cell self-renewal. Adding to the complexity of this issue, non-genomic effects of TH mediated by integrin receptor on cell surface can also modulate several biological functions. Accumulating evidence indicate that regulations by genomic and non-genomic effects of TH overlap. Taken together, these observations suggest that the functions of TH depend largely on cell context, and TH/TR plays a duel role in cancer progression. Therefore, understanding the maze of biological effects of TH has become a necessity when attempting to develop effective therapeutic and preventive strategies in liver cancer.

## Introduction

Thyroid hormone (TH) plays an important role in countless cellular processes, including cell differentiation, proliferation, autophagy, and metabolism (1). The effects of TH on cancer cells are mediated by either genomic or non-genomic signal transduction (2). Deregulation of TH-mediated pathway results in disturbances of cellular functions such as those observed in tumorigenesis (1). An important issue of TH involved in cancer progression is the “TH concentration” in patients (3). Increasing evidence has shown that TH status (hypothyroidism and hyperthyroidism) is involved in the development of several cancers, including ovarian cancer (4), prostate cancer (5), breast cancer (6), and hepatocellular carcinoma (HCC) (7), evidenced by population-based case–control study.

HCC is one of the most common and aggressive human malignancy in the world. Most patients with HCC have an established background of chronic liver disease and cirrhosis. The major risk factors for liver cirrhosis include chronic infection with hepatitis B virus (HBV) and hepatitis C virus (HCV), and alcoholism (8). Cirrhosis develops following long periods of chronic necroinflammation of hepatocytes and is characterized by a decreased capability of hepatocyte proliferation, indicating an exhaustion of the regenerative potential of the liver. Several preclinical investigations have linked TH-mediated response to tumor-suppressing functions or tumor-promoting actions in hepatocarcinogenesis (1).

The main objective of this review is to summarize the basic properties and functional roles of the TH in HCC. In particular, we have encapsulated current knowledge on the contribution of TH-regulated genes that alter cell growth, cell cycle distribution, drug resistance, and metastasis. Hopefully, these pieces of information can provide clues for future anticancer discoveries.

## The Genomic and Non-genomic Actions of TH

**Figure 1:**
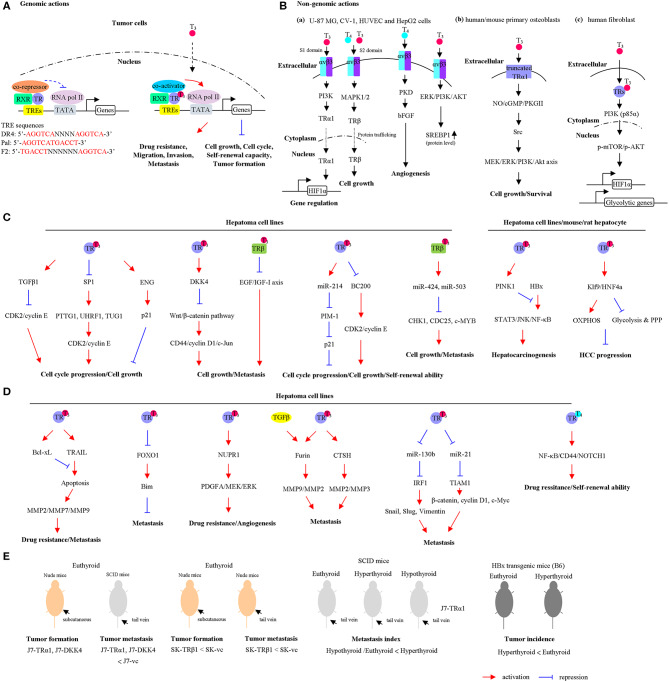
The actions of thyroid hormones (THs) and their corresponding receptors. **(A)** Genomic actions of the TH/thyroid hormone receptor (TR). In the absence or presence of TH, TR/retinoid X receptor (RXR) heterodimers bind to thyroid hormone response elements (TREs). Unliganded TRs associate with corepressors that repress gene transcription. Binding of T_3_ causes corepressor release, coactivator recruitment, and transcriptional activation. **(B)** Non-genomic actions of the TH. (a) The TH interacts with the integrin receptor and activates extracellular signal-regulated kinase (ERK)/phospholipase C (PLC)/protein kinase Cα (PKCα) cascade, which upregulates cell proliferation. U-87 MG cells, glioma cells; CV-1 cells, fibroblast cell lines that lacks functional TRs; HUVEC cells, endothelial cells; HepG2 cells, hepatoma cell line. (b) The T_3_ interacts with the αvβ3 and enhances the phosphatidylinositol 3-kinase (PI3K) pathway, leading to direct trafficking of TRα1 from the cytoplasm to the nucleus and promotes hypoxia-inducible factor-1α (HIF-1α) expression. (c) In the cytoplasm, the liganded TRβ1 associates with the PI3K regulatory subunit, p85α, and activates Akt/mammalian target of rapamycin (mTOR) axis. Consequently, these events regulate gene expression, including HIF1α and glycolytic genes. (d) T_3_ interacts with plasma membrane-associated truncated TRα1 and induces the NO-cGMP-PKGII cascade. Subsequently, this cascade initiates Src/MEK/ERK/PI3K/Akt signaling, which results in induced cell proliferation and survival. The cell lines used in each pathway were shown. The schematic diagram summarizes the findings mentioned in the text including the types of cell lines and animal models. **(C)** TH/TR-mediated cell growth and cell cycle progression via specific signaling pathway are shown. OXPHOS, oxidative phosphorylation; PPP, pentose phosphate pathway. **(D)** TH/TR-mediated cell metastasis, angiogenesis, self-renewal ability, and drug resistance via specific signaling pathway are shown. **(E)** TH/TR-mediated tumor growth and tumor metastasis in murine model are shown. Euthyroid, control; hypothyroid, low T_3_ levels; hyperthyroid, high T_3_ levels. For a detailed description, please see in the corresponding section.

The effects of TH on cancer cells can be divided into two categories: genomic and non-genomic pathways (2). Dysregulation of any of these mechanisms has significant effects on cancer formation and progression. For genomic effect of TH (Figure 1A), T_3_ interacts with thyroid hormone receptor (TR) and other nuclear receptors, such as vitamin D receptor (VDR) and retinoid X receptor (RXR), which in turn regulates transcriptional activities of the target genes (9). TRs are encoded by two separate genes, THRA (TRα) and THRB (TRβ), located on human chromosomes 17 and 3, respectively. The functional domains of TRs are composed of amino terminal A/B domain that may recruit regulatory proteins, DNA-binding domain (DBD), a hinge region that contains the nuclear localization signal, and carboxy-terminal ligand-binding domain that binds to T_3_. TRs function in a ligand-dependent manner via binding to thyroid hormone response elements (TREs) of target gene promoter regions (9). So far, direct repeat (DR4), inverted palindrome (F2), and palindrome (pal) types of TRE have been identified (10). T_3_ binding induces TR conformational changes, causing coactivator [e.g., steroid receptor coactivator 1 (SRC-1)] binding and corepressor [e.g., nuclear receptor corepressor (NCoR) and silencing mediator of retinoic and thyroid receptor (SMRT)] release that lead to chromatin modifications and transcriptional activation (Figure 1A).

For non-genomic effects of TH (Figure 1B), previous studies have shown that the regulatory mechanisms of TH's functions are not totally mediated by nuclear TRs. Other nonnuclear events are involved. It is referred to as non-genomic effects. These reactions are rapid in contrast to genomic effects that occur over minutes or hours and are not involved in gene transcription or protein synthesis. Based on earlier findings, TH (T_4_ and T_3_) interacts with cell surface protein, integrin αvβ3, or plasma-membrane-associated truncated TR or cytoplasmic TR and contributes to regulation of multiple physiological functions (11). In fact, TH interacts with integrin αvβ3 near its Arg–Gly–Asp (RGD)-binding site. Two TH-binding domains of integrin αvβ3 are identified (12). Notably, T_3_ could bind to the S1 domain and activate the phosphatidylinositol 3-kinase (PI3K)/Akt/protein kinase B (PKB) pathway through Src kinase. S1 domain regulates Src and PI3K-mediated TRα translocation from the cytoplasm to the nucleus and enhances target gene expressions such as hypoxia-inducible factor-1α (HIF-1α) (Figure 1Ba) (13). Furthermore, S2 domain mainly recognizes T_4_ and T_3_, consequently activating the mitogen-activated protein kinase/extracellular signal-regulated kinase (MAPK/ERK1/2) pathway via phospholipase C (PLC) and protein kinase Cα (PKCα). Specifically, S2 domain promotes tumor cell proliferation via regulation of nuclear trafficking of TRβ1 from the cytoplasm (Figure 1Ba) (14). Gnoni et al. (15) indicated that translational efficiency of the precursor form of sterol regulatory element-binding transcription factor 1 (SREBP1), which functioned as master regulator of lipid metabolism, was upregulated by T_3_ through activation of MAPK/ERK and PI3K/AKT/mTOR-C1 pathway in HepG2 cells (Figure 1Ba). Notably, these effects were partially abrogated by tetraiodothyroacetic acid (tetrac), which repressed the binding of T_4_ and T_3_ to the integrin αvβ3. These findings suggested that SREBP1-mediated lipid metabolism was regulated by T_3_ via non-genomic effects in hepatoma cell lines (15). In addition, T_3_ interacts with plasma membrane-associated truncated TRα1 and induces the NO-cGMP-PKGII cascade (16). In this TRα-depleted osteoblasts model, re-overepxression of truncated TRα1 partly restored T_3_-mediated effects. Subsequently, this cascade initiates Src/MEK/ERK/PI3K/Akt signaling, which results in induced cell proliferation and survival (Figure 1Bb). Using the human fibroblast-harboring wild-type or mutant TRβ (resistance to thyroid hormones) models, liganded TRβ1 interacts with p85α in the cytoplasm, leading to activating phosphorylation of Akt (Figure 1Bc). Activated Akt promotes the nuclear mammalian target of rapamycin (mTOR)/p70S6K cascade and sequential regulation of several HIF-1α target genes, including glucose transporter 1 (GLUT1), platelet-type phosphofructokinase (PFKP), and monocarboxylate transporter 4 (MCT4) (17–20). Although several non-genomic effects of TH were not observed in hepatoma cell lines, these models have several advantages. For instance, the CV-1, a fibroblast cell line lacking endogenous TR expression, was a good cell line model for observing non-genomic actions of TR (21). Additionally, T_4_ induced angiogenesis through αvβ3/protein kinase D (PKD)/basic fibroblast growth factor (bFGF) in human umbilical vein endothelial cells (HUVECs) (Figure 1Ba) (22), suggesting TH crosstalk with tumor microenvironment via non-genomic actions.

Crosstalk between genomic and non-genomic actions of TH via TR and integrins has been explored. Cao et al. have demonstrated that TH binds to αvβ3 at plasma membrane and controls TRβ1 localization (23). In addition, posttranslational modifications of TRβ1 are regulated by TH/αvβ3. TH treatment triggers MAPK-dependent phosphorylation of TRβ1 at Ser142 (24). This event causes shedding by the receptor of corepressor (SMRT) and recruitment of coactivators that contribute to the transcriptional activity. Understanding how crosstalk occurs between genomic and non-genomic actions to regulate important biological functions will help us design novel drugs or inhibitors to improve treatment of TH-dependent cancers.

## The Impacts of Thyroid Hormone on Cell Growth and Cell Cycle Progression in HCC

Previously, a transplantation model for investigating the effects of TH on hepatocytes was established (25). Small pieces of thyroid tissues were transplanted through the portal vein in to the liver of thyroidectomized rats. After short- and long-term transplantation, the proliferative, synthesize thyroglobulin, and T_3_ release ability were increased. In addition, Columbano et al. (26) group demonstrated that thyromimetic agent GC-1, which was highly selective for interacting and activation of TRβ1 functions, promoted rat hepatocyte and pancreatic cell proliferation. Mechanically, cyclin D1 messenger RNA (mRNA) was upregulated by GC-1 treatment. These findings indicated that TRβ-selective agent GC-1 is an important mitogen for both hepatocyte and pancreatic cells. Work by the same group also showed that T_3_ treatment result in increased incorporation of the DNA replication marker, bromodeoxyuridine (BrdU), in mouse hepatocytes. In addition, treatment with T_3_ and GC-1 has been shown to prevent a spectrum of liver-related diseases such as non-alcoholic fatty liver in rats (27). GC-1 treatment reduced hepatoma cell proliferation and tumor burden through inhibition of p-Met (Y1234/1235), p-ERK1/2, and p-STAT3 pathway (28). Meanwhile, GC-1 treatment did not activate β-catenin and its downstream targets. These findings suggested that GC-1 exerted an antitumor effect in HCC. Transforming growth factor (TGF-β) is induced by T_3_ in HepG2-TR overexpressing cells (29). Moreover, treatment by TGF-β neutralizing antibody but not the control antibody, restored the cell proliferation suppression effect of T_3_. Mechanically, several cell-cycle-related genes such as *CDK2, clyclin E*, and *ppRb* were downregulated by T_3_/TR, while *p21* was induced by T_3_/TR. Notably, suppression of cyclin E, cdk2, and ppRb expression by T_3_ was blocked by treating with TGF-β neutralizing antibody. Pituitary tumor transforming gene 1 (PTTG1) is cell cycle modulator and acts as a securin that suppresses sister chromatid separation and acts as an oncogene implicated in cancer progression (30, 31). Chen et al. demonstrated that *PTTG1* was repressed by T_3_/TR through suppression of transcription factor, SP1 (32). Overexpression of PTTG1 enhanced cell growth and cell cycle progression in Hep3B cells. Notably, the expression levels of PTTG1 and SP1 were inversely correlated with the expressions of TRs in HCC specimens. Endoglin (ENG) is a component of the TGFβ receptor complex that binds to TGF-β1, TGF-β3, activin-A, and bone morphogenetic protein-2 and regulates TGFβ1-mediated responses. *ENG* is directly upregulated by T_3_/TRs in HepG2-overexpressing TR cells (33). T_3_/TR dramatically induced G_0_/G_1_ phase cell cycle arrest. Knockdown of ENG in HepG2-TRα1 cells facilitated p21 polyubquitination and promoted cell growth and cell cycle progression in the presence of T_3_. In addition, antagonization of Wnt signal pathway by Dickkopf 4 (DKK4) was induced by T_3_ in HepG2-TR-overexpressing cells and J7-TRα1 cells (34). Functionally, ectopic expression of DKK4 in J7 cells reduced cell growth and invasion. The effect of DKK4 and TRα1 on tumor growth and metastasis of J7 in animal models was observed without treating T_3_. The tumor growth and metastatic index were repressed by overexpression of DKK4 and TRα1. These observations suggest that TR/DKK4/Wnt/β-catenin axis modulates proliferation and migration of hepatoma cells during metastasis. Dysregulation of epigenetic regulator may contribute to cancer progression (35). Expression of an epigenetic regulator, ubiquitin-like with PHD and ring finger domains 1 (UHRF1), is regulated by TH. The regulatory effect is indirect and mediated by SP1 (36). Inhibition of UHRF1 expression reduced cell growth in hepatoma cell lines via cyclin E/CDK2/p21 axis. Furthermore, overexpression of UHRF1 abolished T_3_-induced cell cycle arrest. Quantitative assessment using clinical samples revealed that expression levels of UHRF1 and Sp1 were elevated in HCC and inversely correlated with TRα1 expression. Another oncoprotein, Stathmin (STMN1), highly expressed in HCC, was directly repressed by T_3_ (37). Depletion of STMN1 inhibited tumor growth *in vitro* and *in vivo*. Additionally, T_3_-mediated cell cycle distributions were attenuated by STMN1. Notably, cyclin A, B, and D levels were reduced in STMN1 knockdown cells. High-throughput screening experiments revealed more specific genes that were directly regulated by T_3_/TR, when chromatin immunoprecipitation coupled with microarray (ChIP-on-ChIP) analysis was performed (38). Among these potential target genes, 304 upregulated and 176 downregulated genes were simultaneously bound by T_3_/TR. A transcription factor, E74-like factor 2 (ELF2), directly downregulated by T_3_/TR, was involved in T_3_-mediated cell growth and cell cycle progression effect. Knockdown of ELF2 in SK-Hep1 cells promoted p21 and p27 expression. Consistent with our results, TH/TR-induced antitumor effects through induction of a differentiation program in the preneoplastic hepatocytes. This view has been supported by experiments using a carcinogen-induced HCC animal model (39, 40). Taken together, T_3_/TR plays tumor suppressor role in regulating cell growth and cell cycle progression (Figure 1C).

Non-coding RNAs (ncRNA) are a class of non-protein coding transcripts frequently dysregulated in various cancers, which also play important roles in biological processes, such as cell proliferation, metabolism, drug resistance, and cell metastasis (41). Expression of a microRNA (miRNA), *miR-214*, was stimulated upon T_3_ treatment (42). PIM-1 is a miR-214 target gene. Furthermore, the effect of T_3_-repressed cell growth was partially restored upon miR-214 knockdown. *MiR-424* and *miR-503* were positively regulated by TH in SK-overexpressing TRβ cell lines (43). Knockdown of those miRNAs reversed the inhibitory effect of TH on CHK1, WEE1, CDC25, c-MYB, and E2F3, in turn impairing the effect of TH on cell growth. A long ncRNA, *BC200*, directly downregulated by T_3_ was identified in hepatoma (44). BC200 enhanced cell proliferation through regulation of cell-cycle-related genes such as cyclin E, CDK2, and p21. Meanwhile, depletion of BC200 repressed the tumor sphere formation frequencies of CD133^+^ subsets of HepG2 and Huh7 cells, compared with control cells. Moreover, the BC200/cyclin E2/CDK2 axis is correlated with poor prognosis in HCC patients (Figure 1C). In contrast, Wang and coworkers demonstrated that T_4_/TRα promoted hepatoma cell, including CSQT-2 and MHCC97-H cells, self-renewal ability through activation of nuclear factor kappa B (NF-κB) and stem cell genes such as CD44, NOTCH1, BMI1, and HIF1α (45). In addition, T_4_ enhanced the percentage of CD90-positive HCC cells and drug resistance. In this model, T_4_ acted as promoter of CSC self-renewal (Figure 1D). Although T_4_ is predominantly secreted from the thyroid gland, the affinity of the TR for T_4_ is lower than that for T_3_. Different treatment (T_4_ or T_3_) may trigger different signaling pathways via genomic or non-genomic actions and results in controversial results. Other studies reported that TH/TRs promoted cell growth in other cancer cell types, including glioma (46), breast (47), and prostate cancer (48). Kress *et al*. (49) demonstrated that overexpression of TRα1 and activation of Wnt/β-catenin pathway promoted colorectal cancer formation. TRα1 enhanced tumorigenesis in APC^+/1638N^ mice with a Wnt-activated genetic background. Notably, a study reported that thyroid status regulated tumor formation and metastasis in experimental animal models and humans (50). Lin et al. (51) reported that mutated TRs in human HCCs and cultured cells lose its T_3_ binding and transcriptional activities and subsequently exhibit dominant-negative activity. Another evidence demonstrated that mutation form of TRs isolated from HCC patients (HCC-TR) were transfected into HepG2 cell lines (52). Moreover, differential expressed genes in those cell lines were measured using microarray analysis. Interestingly, only a subset of genes regulated by HCC-derived TR mutants was also regulated by the corresponding wild-type TRs. Furthermore, the mutation form of TR could regulate additional target genes not modulated by the wild-type receptors. Due to the complexity of these effects, TH/TR could play a tumor-suppressive and/or protumorigenic role in a cellular context-dependent manner.

## The Impacts of Thyroid Hormone on Autophagy in HCC

Hepatitis B virus X protein (HBx) encoded by the X gene of the HBV is involved in tumor formation, apoptosis, drug resistance, and metastasis through various pathway (53). By treating with or without T_3_ in HBx-transgenic mice, T_3_ could protect hepatocytes from HBx-triggered cell damage through regulation of mitochondrial quality control *in vivo* (54). PTEN-induced kinase 1 (PINK1) is induced by T_3_ *in vitro* and *in vivo*. PINK1 can activate and recruit Parkin, an E3 ligase, to enhance HBx protein ubiquitination and trigger selective mitophagy (Figure 1C). Furthermore, Chi *et al*. demonstrated that TH treatment repressed liver carcinogenesis through activation of autophagy (55). Death-associated protein kinase 2 (DAPK2) expression was positively regulated by T_3_. In a murine model, overexpression of DAPK2 attenuated diethylnitrosamine-induced toxicity and DNA damage via promotion of autophagy. These findings suggest that TH treatment can be an effective therapeutic option for liver cancer.

## The Impacts of Thyroid Hormone on Drug Resistance in HCC

Drug resistance is a complex process, with many intrinsic and extrinsic factors regulating the resistance potential of HCC cells (56). In addition, drug resistance is a well-known nightmare when cancers and diseases become tolerant to drug treatments. Although several types of cancers are initially susceptible to anticancer treatment, over time, they can develop resistance through mechanisms such as increased tumor heterogeneity, alteration of tumor microenvironment, and renewal of cancer stem cells. Several studies demonstrated that T_3_ leads to promotion of anticancer drug resistance. These associations between TH/TR and drug resistance are shown in Figure 1D. Chi and coworkers demonstrated that tumor necrosis factor (TNF)-related apoptosis-inducing ligand (TRAIL) was directly upregulated by T_3_ in TR-overexpressing HepG2 and J7 cells (57). In those cell lines, T_3_ suppresses r-TRAIL and chemotherapeutic drugs-induced apoptosis. Several experimental results demonstrated that T_3_-induced Bcl-xL, an antiapoptotic gene, prevented hepatoma cells from TRAIL-induced apoptosis, in turn promoting hepatoma cell metastasis. Another report indicated that *Bim*, a proapoptotic gene, was indirectly regulated by T_3_ (58). Furthermore, Bim was directly regulated by Forkhead box protein O1 (FoxO1), which was repressed by T_3_ through genomic and non-genomic effects. Of note, T_3_-repressed Bim prevented hepatoma cells from cisplatin, doxorubicin, and TRAIL-induced apoptosis. Nuclear protein-1 (NUPR1) was positive regulated by T_3_/TR through direct binding to the−2066/-1910 region of the NUPR1 promoter (59). Ectopic expression of NUPR1 promoted angiogenesis through the regulation of PDGFA/MEK/ERK signaling cascade. Furthermore, TR/NUPR1 axis enhanced sorafenib resistance in Mahlavu and Huh7 cell lines *in vitro* and *in vivo*. In addition, Wang *et al*. reported that T_4_ enhanced drug resistance of CSQT-2 and MHCC97-H cells in a TRα-dependent manner (45). Previous studies reported that TH, including T_4_ and T_3_, induced P-glycoprotein (P-gp) gene expression, which is also known as multidrug resistance protein 1, and plays a significant role in multidrug-resistant phenotype (60–62). Accordingly, TH/TR manifests an oncogene-like effect via increasing angiogenesis and drug resistance in hepatic tumor microenvironment.

## The Impacts of Thyroid Hormone on Cancer Cell Metastasis in HCC

The process of cancer metastasis remains an important issue when it comes to successful management of malignant diseases (63). Martinez-Iglesias et al. (64) group indicated that TRβ1 acts as a suppressor of tumor metastasis in hepatoma (SK-Hep1) and breast cancer (MDA-MB-468) cells. Ectopic expression of TRβ1 in those cells inhibited tumor metastasis, both *in vitro* and *in vivo*, validating a suppressor role of TRβ (Figure 1C). This study showed that TRβ1 had major suppressive effect on metastasis. Furthermore, the authors showed that double-knockout thra thrb mice are induced to generate epithelial tumors, suggesting that TR deficiency could inhibit tumor formation at early stages while enhancing cancer progression at later stages in the skin carcinogenesis protocol. The same group has also demonstrated that hypothyroidism could retard tumor formation and promote metastasis independent of TRβ1 expression (65), suggesting that the influence of thyroid status in HCC progression is more significant than that of the TRβ1-mediated effects. In addition, T_3_-mediated *Furin* upregulation cooperated with TGF-β1 signaling pathway to regulate cellular function (66). Ectopic expression of Furin promoted cell migration, invasion, and metastasis *in vitro* and *in vivo* (Figure 1D). In addition, an oncomiR, *miR-21*, was upregulated by T_3_ in HepG2-TR cell lines (67). Inhibition of miR-21-induced Hep3B-TRα1 cell migration was rescued by T_3_ treatment. Meanwhile, T-cell lymphoma invasion and metastasis 1 (TIAM1) was a potential target of miR-21 and downregulated by T_3_. Quantitative assessment using clinical samples showed that miR-21 was positively correlated with TRα1 in HCC specimens (Figure 1D). On the other hand, *miR-130b* was negatively regulated by T_3_/TR (68). Cell migration was enhanced by T_3_ but partially repressed upon miR-130b overexpression. Notably, miR-130b regulated cell migration, invasion, and metastasis, which was mediated by interferon regulatory factor 1 (IRF1) (Figure 1D). Hyperthyroidism in J7-TRα1 xenograft models promotes cell metastasis compared to eu- and hypothyroid. T_3_/TR, miR-130b, IRF1, and EMT-related genes axis regulated the motility and invasion of hepatoma cells. Thus, these studies suggest that T_3_/TR exerts an oncogene-like effect in metastasis of liver cancer. Due to different experimental approaches, overexpression of TRs in J7 and SK-Hep1 cells under normal physiological concentration of TH (euthyroid) suppressed tumor metastasis in nude and severe combined immunodeficient (SCID) mice (Figure 1E). However, it has the opposite effects by treating T_3_ (hyperthyroid/hypothyroid vs. euthyroid group) in xenograft model by tail vein injection method (Figure 1E). In addition, TH suppressed HBx-induced hepatocarcinogenesis in a transgenic mice model. The different genetic background (immune vs. immunodeficient mouse model) maybe responsible for the conflicting effects of TH/TR in HCC progression. On the other hand, tumor microenvironment can modulate tumor formation, metastasis, drug resistance, and therapeutic response. Stromal cells orchestrate the microenvironment and provide specific molecules such as growth factors and proangiogenic factors to facilitate tumor cell survival and metastasis (69). T_3_ enhanced phosphorylation of GSK-3β and Akt in TRα1-overexpressing endothelial cells through non-genomic actions (70). The crosstalk between cancer cell and stromal cells may modulate the function of tumor suppressors, resulting in a switch to promote oncogenesis.

## The Impacts of Thyroid Hormone on Metabolism in HCC

Previous experiments by our group showed that metabolism-related genes, including aldo-keto reductase family 1, member b1 (AKR1B1) (71), methionine adenosyltransferase 1a (MAT1A) (72), and chromosome 19 open reading frame 80 (C19orf80) (73), upregulated by T_3_ in TR-overexpressing HepG2 cells, modulated HCC progression. In particular, Tseng and coworkers demonstrated that TH/TR regulates lipid metabolism via C19orf80-triggered autophagy pathway (73). Multiple lines of evidence, including ours, indicated that hepatic lipid turnover rate was induced by TH treatment. Major risk factors such as non-alcoholic fatty liver disease (NAFLD) and metabolic syndrome for HCC, especially for non-hepatitis B and non-hepatitis C HCC (NBNC-HCC), have been reported (74). Recently, it was found that *TUG1*, responsible for regulating glycolysis and metastasis, was downregulated by T_3_/TR in HepG2-TR cells (75). Depletion of TUG1 reduced cell cycle progression and soft agar colony formation (Figure 1C). Notably, α-fetoprotein was partially downregulated by T_3_/TR through suppression of TUG1. By analyzing clinical samples, mRNA expression levels of AFP were strongly and positively correlated with TUG1 and associated with unfavorable prognosis in patients with NBNC-HCC. Kowalik et al. (76) demonstrated that T_3_ treatment in HCC-bearing rats dramatically repressed the number and burden of HCC. Notably, T_3_-induced metabolic reprogramming (switch from glycolysis to oxidative metabolism) precedes preneoplastic nodules regression (Figure 1C). The identified metabolites are the intermediate products of the responsible metabolic processes. Further study is mandatory to determine the oncometabolites regulated by TH/TR to overcome the current limitations of HCC therapy.

## Clinical Significance of Thyroid Hormone and Its Receptor in Liver Cancer

A case–control study reported that long-term hypothyroidism was associated with significantly elevated risk of HCC, independent of other major HCC risk factors (7). In particular, this finding was statistically significant only among women. Subsequently, the correlations between thyroid functions, clinical parameters, and survival functions were investigated by Pinter et al. (77). They found that thyroid-stimulating hormone (TSH) and free T_4_ (fT_4_) levels were highly correlated with prognostic factors such as tumor size and C-reactive protein. Notably, high serum fT_4_ levels were independently correlated with poor prognosis in HCC. Chu et al. demonstrated that higher levels of TSH and fT_4_ were independently associated with favorable progression-free and overall survival in HCC patients (78). However, the outcome associations were opposite between advanced HCC patients treated by sorafenib and systemic chemotherapy. In addition, truncating and missense mutations of TRs were identified in HCC specimens and HCC cell lines. Of note, v-erbA is a mutant form of TRα with disrupted T_3_-mediated regulation functions for genes such as follistatin, activin βC, thrombomodulin, N-myc downstream-regulated gene 2, Sine oculis-related homeobox-1 homolog, and Ras-GTP-releasing protein 3, in turn leading to liver carcinogenesis in a transgene mouse model (79). Our previous study has shown that thyroid-stimulating hormone receptor (TSHR) is highly expressed in HCC and associated with unfavorable survival outcomes (80). Meanwhile, mutations of TSHR in exon 10 were identified in HCC specimens, but not in the adjacent non-cancerous tissues, suggesting a growth advantage of tumor cells harboring such mutations.

## Conclusions

Based on these findings, the distinct functions of TH/TR in HCC depend on TH (T_4_ vs. T_3_), TRs expression status (mutation/different isoforms), integrin αvβ3, coregulators, cell lines, cell context, and cancer stage. Liver cancer is a complex experimental model when it comes to exploring the relationship between the TH/TR and tumor progression. In the current article, the T_3_/TR-regulated genes in hepatoma cell lines, involved in regulations of tumor growth, cell cycle progression, drug resistance, and metastasis, are comprehensively shown in Figure 1 and listed in Table 1. Although several protein-coding genes and ncRNAs are known to be regulated by T_3_/TR, existing knowledge regarding the key signaling pathways responsible for hypothyroidism-related liver carcinogenesis is incomplete. The collective results support distinct associations between the target genes and TH/TR networks that regulate cancer cell growth, anticancer drug resistance and metastasis. In addition, these associations could be different depending on cancer types. Understanding the details of these cross-talks is therefore essential to successfully identify novel therapeutic targets.

**Table 1:** Thyroid hormone (TH)/thyroid hormone receptor (TR)-regulated genes and their potential mechanisms in hepatocellular carcinoma (HCC).

**Gene name**	**Principal functions**	**Molecules and signaling pathways involved^a^**	**HCC development**	**Prognostic markers in HCC^b^**	**Up- or downregulation^c^**	**Cell types**	**References**
TGFβ	Cell growth Cell cycle	Cyclin E, CDK2, p21	Dual role	✓	Up	HepG2-TR	(29)
PTTG1	Cell growth Cell cycle	SP1	Progression	✓	Down	HepG2-TR cells, Huh7, J7, Hep3B, Mahlavu	(32)
Endoglin	Cell growth Cell cycle	P21 ubiquitination	Regression	✓	Up	HepG2-TR, J7	(33, 81)
DKK4	Tumor formation Migration Invasion	Wnt pathway, CD44, cyclin D1, MMP2, MMP9	Progression	✓	Up	HepG2-TR, J7-TRα1, J7	(34)
PINK1	Tumor formation DNA damage ROS Mitophagy	LC3, Parkin, HBx ubiquitination	Regression	✓	Up	HepG2-TR, HepG2	(54)
DAPK2	Tumor formation Autophagy	P62	Regression	✓	Up	HepG2-TR, CL-48-TRα1, HepG2	(55)
ELF2	Cell growth	P21, p27	Regression	–	Down	HepG2- TRα1, J7-TRα1, HepG2	(38)
miR-214	Cell growth Tumor formation Apoptosis Angiogenesis	PIM1, HDGF	Regression	✓	Up	HepG2-TR, Huh7, SK-Hep1	(42, 82)
miR-424 miR-503	Proliferation Invasion	CHK1, WEE1, CDC25, c-MYB, E2F3	Regression	✓	Up	SK-TRβ, HepG2-TRβ, MDA-MB-468, MDA-MB-468-TRβ	(43, 83, 84)
UHRF1	Cell growth Tumor formation	cyclin E, CDK2, p21	Progression	✓	Down	HepG2-TR, HepG2, Huh7, J7, Hep3B, Mahlavu, SK-Hep1	(36)
STMN1	Cell growth Tumor formation	Cyclin A, cyclin B, cyclin D	Progression	✓	Down	HepG2-TR, HepG2, J7	(37)
BC200	Proliferation Tumor growth Stem cell-like phenotype	P21, p27, CDK2, Cyclin E2	Progression	✓	Down	HepG2-TR, HepG2, Huh7, Hep3B, J7, SK-Hep1	(44)
TRAIL	Drug resistance Metastasis	Bcl-xL, caspase-3, caspase-8, MMP2, MMP9	Progression	✓	Up	HepG2-TR, J7-TR, HepG2, Huh7, J7,	(57)
Bim	Apoptosis Drug resistance	FOXO1, Akt, NF-κB, MiR-200a	Progression	–	Down	J7-TR, Hep3B-TR, HepG2, Huh7, Hep3B, J7, Mahlavu, SK-Hep1	(58)
NUPR1	Proliferation Migration Tube formation Drug resistance	ERK/HIF-1α/p70S6K/VEGFA	Progression	✓	Up	HepG2-TR, J7-TR, HepG2, Huh7, J7, Mahlavu, HUVEC	(59)
miR-21	Migration Metastasis	TIAM1, β-catenin	Progression	✓	Up	HepG2-TR, Hep3B-TRα, HepG2, Hep3B, SK-Hep1	(67)
miR-130b	Invasion Metastasis	IRF1, EMT-related genes, p-STAT3, p-AKT	Regression	✓	Down	HepG2-TR, J7-TR, HepG2, Huh7, J7, Mahlavu, SK-Hep1	(68)
KLF9 HNF4	Differentiation Glycolysis pentose phosphate pathway	Metabolic-related genes	Regression	✓	Up	HepG2, Mahlavu	(76)
TUG1	Cell growth Cell cycle Senescence	Cyclin E, CDK2, p27, AFP, EZH2	Progression	✓	Down	HepG2-TR, Hep3B, SK-Hep1	(75)

a *Downstream molecules and signaling pathways involved in target gene-mediated functions*.

b *✓: Target gene acts as a prognostic marker in HCC; –: information is unavailable*.

c *Up: target gene was upregulated by TH; down: target gene was downregulated by TH*.

## Author Contributions

Y-HL: writing—original draft preparation. K-HL and C-TY: writing—review and editing. C-TY: supervision.

## Conflict of Interest

The authors declare that the research was conducted in the absence of any commercial or financial relationships that could be construed as a potential conflict of interest.
